# Acknowledgment to the Reviewers of *Brain Sciences* in 2022

**DOI:** 10.3390/brainsci13020169

**Published:** 2023-01-19

**Authors:** Brain Sciences Editorial Office

**Affiliations:** MDPI AG, St. Alban-Anlage 66, 4052 Basel, Switzerland

High-quality academic publishing is built on rigorous peer review. *Brain Sciences* was able to uphold its high standards for published papers due to the outstanding efforts of our reviewers. Thanks to the efforts of our reviewers in 2022, the median time to first decision was 17 days and the median time to publication was 39 days. Regardless of whether the articles they examined were ultimately published, the editors would like to express their appreciation and thank the following reviewers for the time and dedication that they have shown *Brain Sciences*:
A. N. M. Mamun-Or-RashidKeith RochfortAarti NagayachKeith SchneiderAbadie MarlèneKelly WhitefordAbbas F. AlmullaKen WalderAbbas JarrahiKeng Siang LeeAbbas OrandKenneth CiuffredaAbdel-Nasser SharkawyKenneth HandelmanAbdolkarim HosseiniKentaro HiromitsuAbdulkarim HasanKepinska OlgaAbdullah Md. SheikhKerri Lynn SchellenbergAbhimanyu ThakurKe-Vin ChangAbhishek KumarKevin DarbyAbijeet MehtaKevin P. UribeAbraham PouliakisKevin PatersonAchim SauerKim BulkeleyAchmad RizalKim Ryun DrasbekAdam B. EdwardsKimberly R. ByrnesAdam HamedKimbra KenneyAdam ReevesKin Israel NotarteAdam WiśniewskiKiran SapkotaAddisson SalazarKlaus KirchhofAditya PawarKlaus PetryAditya SinghKlearchos PsychogiosAdnan KhanKoji NishimuraAdolfo GarciaKonrad A. SzychowskiAdrià ArboixKonrad AumannAdriana Caldo-SilvaKonrad Leszek MendralaAdriana GrigorașKonstantin SlavinAdrienne RomerKonstantin TziridisAfrouz Azari AndersonKonstantinos DrossosAfshin ShoeibiKostas MichalopoulosAftab AlamKouko TatsumiAfzal MisraniKoya YamashiroAgata Ando'Kristian RotaruAgata Białecka-DębekKristóf Endre KárolyAgata GabryelskaKunal VakhariaAgata GozdzKun-Eek KilAgata MarjańskaKuo-Liang HuangAgnese ZazioKurt A. JellingerAgnieszka J. SzczepekKurt SteinmetzgerAgnieszka PlutaKwadwo Appiah-KubiAgnieszka PregowskaKwun Kei NgAgnieszka RynkiewiczKyle BurghardtAgnieszka ŚliwkaKymberly YoungAgnieszka WąsikKyung-hun KimAgnieszka WolskaLachlan KentAgustina BirbaLadan Ghazi SaidiAhmad M. OsailanLaila CraigheroAhmed BahgatLang ChenAhmed ElnakibLara J. PierceAhmed NegidaLari M. KoponenAida De Arriba-ArnauLarry AbelAikaterini FitsioriLassina BarroAinhoa AlberroLaura AngiolettiAitor Garay-SánchezLaura BaroncelliAjay PalLaura GuerreroAkash GuruLaura SpinuAki TsuchiyagaitoLaura TascónAkihiro IshiiLaura V. SchaeferAkio KimuraLaura W. WesseldijkAl Baraa Abdulrahman Al-MekhlafiLauren SchmittAlan PalmerLauren StewartAlan WislerLauryn R. ZipseAlanna StrongLaxmi T. RaoAlbert Van Der ZwanLéa PilletteAlberto CacciolaLea ScherschinskiAlberto DelaidelliLeandro R. D. SanzAlberto Quílez-RobresLeanne ElliottAlberto SardellaLee BartelAldo Alberto ContiLee D. CranmerAleix SolanesLee GilmanAlejandro Botero-CarvajalLee-Kuen ChuaAlejandro Rodríguez-VázquezLeen CatrysseAlejandro Samhan-AriasLeif SimmatisAleksandar MiladinovicLena K. L. OestreichAleksander ChaibiLeo PruimboomAleksandra Gilis-JanuszewskaLeo VeenmanAleksandra Gruszka-GosiewskaLeonard AbbedutoAleksandra IchkovaLeonardo LopianoAleksandra JasielskaLeonardo ManzariAleksandra K. Eberhard‐MoscickaLeonardo TariciottiAleksandra Karczmarska-WódzkaLeonhard SchilbachAleksandra KristoLevente KapásAleksandra SzczepkowskaLiane KaufmannAleksandra Wisłowska-StanekLiang ZhangAleksey ZaitsevLiang-Jen WangAlessandra BortoluzziLidia PerencAlessandra ConsonniLidia SousaAlessandra CostanzaLihong HaoAlessandra FanciulliLilach SoreqAlessandra FiorettiLilah TokerAlessandra LintasLilia Y. KucheryavykhAlessandra Maria VitaleLiliana AlbertazziAlessandra P. PrincivalleLiliana Ibeth Barbosa-SantillanAlessandra SaporitiLin ZhangAlessandra VergallitoLin ZhuAlessandro CaffòLina PalaiodimouAlessandro GuidaLinda AngellAlessandro PedicelliLinda HersheyAlessandro PincherleLindsay McCaryAlessandro PoggiLinn NorbomAlessandro SalvalaggioLipaz Shamoa-NirAlessandro StefaniLirong WangAlessandro TonacciLisa LandgrafAlessandro VittoriLisa M. GallagherAlessandro ZampognaLisa M. OakesAlessia CarboniLiubov GorbachevaAlessia GallucciLiubov TkachevaAlessia SurgoLiudmila LegostaevaAlessio FacchinLivia PetrescuAlessio ToraldoLivius V. D'UscioAlex KieferLi-Wei ChouAlex Martino CinneraLiziane BouvierAlex P. JamesLjubica KonstantinovićAlexander DimitrovLonglong TuAlexander DityatevLoredana RacitiAlexander GoettkerLorena Elena MeliţAlexander J. PoplawskyLorena Joga-ElviraAlexander KaltenboeckLorena Martín-AguilarAlexander KlothLorenzo BrognaraAlexander LogemannLorenzo MagrassiAlexander N. SavostyanovLorenzo TonettiAlexander RitterLouisa Marguerite ChristensenAlexander Yu MeigalLouise BeattieAlexandra EconomouLourdes Martinez-NietoAlexandra FietsamLubova RenemaneAlexandra JesseLuca BruschiniAlexandra LatineLuca CavaggioniAlexandre A. GuerinLuca PetrignaAlexandre Gonzalez-RodriguezLucia MencarelliAlexandre MoreiraLuciano FurlanettiAlexandrina S. NikovaLucília CardosoAlexandru Silvius PescariuLucio VellosoAlexey BoykoLucy TroupAlexey SarapultsevLudmila MáčováAlexis Moscoso RialLudmiła Zając-LamparskaAlfonsas VainorasLuigi La ViaAlfonsina D'IorioLuigi TaraniAlfonso MastropietroLuigi TesioAlfonso Salgado RuizLuis Exequiel IbarraAlhamzawi RahimLuis Filipe Moutinho LeitãoAli BahadurLuis Jaime Castro-VegaAli BayramLuis RiquelmeAli CaykoyluLuis RodrigoAli FarzamniaLuis Suso-MartíAli KhademLuis Villalobos-GallegosAlice WitneyLuisa BarrosAlina CernasevLuisa LorenziAlina WilkowskaLuisa Losada PuenteAlon SinaiLuisa María BotellaAlvaro Llorente-BerzalŁukasz A. PoniatowskiAlvaro Otero RodríguezŁukasz M. MilanowskiAlyson J. ChroustŁukasz RzepińskiAlyx TaylorLuke J. NeyAmad ZafarLuke NormanAmalesh GopeLuminita NicolescuAmalia CorneaLut TamamAmalia Floriou-ServouLydia Giménez-LlortAmanda Barkley-LevensonLydia Yahia-CherifAmany SholkamyM. Quadir SiddiquiAmar PatelM. S. ZobaerAmaranta De Miguel-RubioMaciej DaniszewskiAmarinder Singh SinghMaciej SikoraAmarish YadavMadhubanti MullickAmaya Epelde-LarrañagaMagdalena Eriksson DomellöfAmbarish PawarMagdalena GębskaAmelia Franke-RadowieckaMagdalena Jastrzębska-WięsekAmir Hossein MehrsafarMagdalena KoszewiczAmir Kaywan AftahyMagdalena Krbot-SkoricAmirmasoud AhmadiMagdalena Markowicz-PiaseckaAmol S. PatwardhanMaggie GuyAmy M. MasnickMahar FatimaAmy R. BlandMahendran SekarAna AdanMahmoud AbdallatAna Carolina De Sousa SilvaMahmoud ElbattahAna Luiza Costa ZaninottoMahrous Abdelbasset IbrahimAna Manzano-LeónMaja Rogić VidakovićAna María Navarro IncioMakella S. CoudrayAna Rita VazMaki KubotaAna SantosMalcolm R. BattinAnabela CorreiaMalena Daich VarelaAnabela Cruz-SantosMalgorzata BurekAna-Isabel Corregidor-SánchezMałgorzata KujawskaAnalía BortolozziMałgorzata MoszyńskaAnalìa L. ArévaloMałgorzata StachońAnamaria JurcauMalik AydinAnan SrikiatkhachornMamede De CarvalhoAnanda SidartaMa'mon M HatmalAnastasiya LopukhinaMamoru ShibataAnastassia ZabrodskajaMan GaoAnbazhagan SathiyaseelanManabu NatsumedaAnca ZanfirescuManikandan PanchatcharamAnders J. ǺgmoManikantam GaddamAndleeb KhanManindra TiwariAndrás Bilkei-GorzóManish DhawanAndré MaierManish DwivediAndre RodackiManish RanjanAndrea AgugliaManishkumar PatelAndrea AlbonicoMannes PoelAndrea AlexandreMansour HaddadAndrea BergerManuel A. SchmidtAndrea CaraiManuel Anglada-TortAndrea De GiacomoManuel PontesAndrea FabboManuela LeriAndrea M. AlexandreManuela PaechterAndrea MarcinnòManuela Pastore-WappAndrea MariniMara BellatiAndrea MininiMarawan Abd ElbasetAndrea RegnerMarc JacobsAndrea RussoMarc JeschkeAndrea TacchinoMarc LochbaumAndrea TinnirelloMarc VidalAndreas GantenbeinMarcel NaumannAndreas IhleMarcel Olde RikkertAndreas KalckertMarcello MocciaAndreas KoupparisMarcin SiwekAndreas KyrozisMarcin SochalAndreas Meyer HeimMarco Aurelio M. FreireAndreas NowackiMarco BertaminiAndree HartantoMarco BoAndrei C. HolmanMarco BonifatiAndrei DragomirMarco BortolatoAndrei SurguchovMarco BruschiAndrej M. SavićMarco CambiaghiAndrés Calvillo-TéllezMarco CavalloAndrés Da Silva CandalMarco IosaAndrés Gené SampedroMarco Matteo CicconeAndrew B. StergachisMarco PrenassiAndrew Chih Wei HuangMarco RivaAndrew ClarksonMarco V. CorniolaAndrew E. LittmannMarcos Eduardo ScheicherAndrew HooymanMarcos Mecías-CalvoAndrew J. KobetsMarcus JeschkeAndrew J. MilneMarcus VetterAndrew LarnerMarek LapkaAndrew Stuart GibbonsMarek MotykaAndrew TolmieMargalida Coll-AndreuAndrey V. DubovoyMargaret M. StrattonAndrzej PiotrowskiMargarida Duarte AraújoAngela CarusoMargarita ZachariouAngela ComanducciMargarita ZaytsevaAngela LombardiMargherita NeriAngelique M. BlackburnMargit PissarekAngelo BellinviaMarguerite TernesAngelo CarneiroMaria AlievaAngelo RegaMaría Antonia Parra-RizoAngelo TorrenteMaria BogdanAni GasparyanMaria BoveAniello MaieseMaria Carmen Martin BuroAnil AnnamneediMaría CarrataláAnil Kiran ChokkallaMaria CotelliAnindya GangulyMaría Dolores García-HernándezAnita KovácsMaria Elena Cuartero-CastañerAnja KräplinMaria Elide VanutelliAnjali SivaramakrishnanMaria Elsa GambuzzaAnjolii DiazMaría Florencia PignataroAnke HölligMaria Giovanna BiancoAnn D. DowkerMaria Giuseppina BartoloAnna B. VolnovaMaría GómezAnna CieślińskaMaria Isabel García-PlanasAnna Drelich-ZbrojaMaria K. JonsdottirAnna FiveashMaria Luisa BringasAnna HadjihambiMaria MeringoloAnna KamzaMaría Orosia Lucha-LópezAnna Lena BielMaria Paola MogaveroAnna LewandowskaMaría PérezAnna Maria Birkl-TöglhoferMaria RubegaAnna N. BukiyaMaria SofologiAnna OlczakMaría Teresa Iglesias LópezAnna PecchinendaMaria Teresa InzitariAnna SulekMaria Teresa RestivoAnna TirkkonenMaria V. TurovskayaAnnabel D. NijhofMaría Verónica BaezAnna-Lisa SchulerMariagiovanna CantoneAnnamaria PetitoMariagrazia RossiAnne HoffmannMarian SauterAnne-Cécile BoulayMariana NucciAnnegret HabichMarianna S. TsataliAnneli G. OzanneMarianna StellaAnnie SwanepoelMarianne ChapleauAnselm B. M. FuermaierMarie-Lise Jaffrain-ReaAnselmo CaricatoMarie-Louise ElkjaerAnthony SuraceMarie-Michele BriandAntje Büttner-TeleagăMariko KawashimaAntje MefferdMariko SaitoAntoine LangeardMarina BuzziAntoine ShahinMarina KhodanovichAntonella CaccamoMarina Kleopatra BozikiAntonella CasamassaMarina LaganaroAntonella LettieriMarina M. SchoemakerAntonella Ragnini-WilsonMarina PavlovaAntonello PennaMarina Varela DuránAntonia CianciulliMarina Zaric KonticAntonia MeyerMario Alberto García RamírezAntonino GermanaMario DalmasoAntonino ManiaciMario LucianoAntonio AmmendoliaMario ŠtefanovićAntonio BrunoMario TranfaAntonio CicchellaMarion RappAntonio D'AmatiMarios G. KrokidisAntonio Flores-BurgessMarius BirleaAntonio GiordanoMarius YamakouAntonio Ivano TriggianiMariyana SchoultzAntonio LeoMark A. StokesAntonio MaffeiMark EldridgeAntonio MelcarneMark FelixAntonio NarzisiMark H. MyersAntonio Quintero-RincónMark IsraelAntonio RomanoMark QuiggAntonio ValentínMark ReybrouckAnuenue Baker-KukonaMark Stephen MennemeierAnupa Ambili VijayakumariMark W. MillerAparna RaghuramMark WallaceApostolos GkatzionisMarkku PartinenArdalan AarabiMarko ŽivanovićAreej AlwabilMarkus AswendtArjen AlinkMarkus FendtArmando CaseiroMarkus J. RantalaArmin SchniderMarkus RiesslandArnab BanerjeeMarta BienkiewiczArnaud BadetsMarta Del ÁlamoArnaud GouelleMarta Dziedzicka-WasylewskaArryn A. GuyMarta GhioArtur KrzyżakMarta GrodzikArtur PałaszMarta KaminskaArtur Schumacher-SchuhMarta KopańskaArtur SłomkaMarta Marszalek-GrabskaArtyom ZinchenkoMarta Morgado CorreiaAsanka BulathwattaMarta Pérez-de-Heredia-TorresAshesha MechineniMarta RusnakAshok IyaswamyMarta ValenzaAshutosh TripathiMarta Vergara-MartínezAsli AykacMarta Waliszewska-ProsółAssef ZareMartijn BeudelAthanasia PrintzaMartin BogdanAthanasios KoutrasMartin KlietzAthanasios N. TsartsalisMartin PienkowskiAtiah H. AlmalkiMartin ProescholdtAude RobertMartin RakusaAurel PeraMartin ZabackAurélie LedreuxMartina LickováAurore ThibautMarvin Antonio Soriano-UrsuaAuwal AbdullahiMarwa IbrahimAvishai HenikMary Anna VenneriAxel HuttMaryam TofangchihaAyataka FujimotoMarzia SegùAyman W. El-HattabMasahiro OkamotoAysegul YildizMasahisa KatsunoAytaç AltanMasanobu IbarakiAytuğ OnanMasaru TanakaAyumu YamashitaMasoumeh Kourosh AramiAzlina Binti Mohd KosninMassimiliano ConsonBabak BakhshinejadMassimiliano ToscanoBachir KassasMassimo Di GraziaBai Chuang ShyuMassimo GrassiBalamayooran TheivanthiranMassimo TusconiBalapal BasavarajappaMateusz BujkoBarbara CalabreseMateusz GrajekBarbara KosmowskaMateusz ŁucBárbara Paranhos CoelhoMathieu Di MiceliBarry D. WaterhouseMathilde Hauge LercheBarry ReynoldsMatteo Alessio ChiappediBarry SetlowMatteo Angelo FabrisBartłomiej GmajMatteo Bruno LodiBarzykowski KrystianMatteo CarpiBasanagoud D. MudigoudarMatteo ChiappediBasant K. PuriMatteo Cotta RamusinoBashayer R. Al-MubarakMatteo FeurraBasheer MannasahebMatteo GuidettiBastian SajonzMatteo NeriBeata Sarecka-HujarMatteo PaciniBéatrice C. DurandMatteo RipaBeatrice Gabriela IoanMatteo SpezialettiBebiana CondeMatthew C. TateBehnam RashidiehMatthew E. HirschtrittBehnam VafadariMatthew GardnerBehzad IravaniMatthew HoptmanBehzad KhorashadMatthew J. O'BrienBei ZhangMatthew McCordBelgin SeverMatthew Q. MillerBen Abdallah IsmailMatthew SacchetBence RaczMatthias GriederBenedito C. PrezotoMatthias ProostBeniamina MercanteMatthias SchmitzBenita WiatrakMattia BarbotBenjamin GreenwoodMattia DoroBenjamin H. DurhamMaura M. E. PilottiBenjamin VoellgerMauricio Adolfo Ramírez-MorenoBerdien W. Van Der LindeMaurizio MarchesiniBernadette MurphyMauro AdenzatoBernardo LopezMax SarmetBertil RydenhagMaximilian J. MairBeth FisherMayur B. KaleBethany SussmanMd Daud Mohd KhairiBetty C. SolivenMd Jobair Hossain FarukBhagyashree JoshiMeco GiuseppeBhagyashri BurguteMeekyung HanBharat MishraMeenakshisundaram BalasubramaniamBianca Maria Serena InguscioMeera SoundararajanBijar GhafouriMeg E. MorrisBirgit LudwigMeghan E. MartzBirgitta Dresp-LangleyMehmet KayaBiswajit MaharathiMehmet MahmutBo HuMehrdad DadgostarBochao ZouMehrnaz ShoushtarianBogdan IliescuMehwish AnwerBogusław TwarógMeixue DongBojana MilutinovicMeiyan WangBoon Giin LeeMelanie BoltzmannBoris BurleMelek OzturkBrach PostonMelike KahyaBrady S. DeCoutoMelissa D. StockbridgeBrandon Lucke-WoldMelissa NassifBrian A. ZaboskiMelvin BergerBrian D. AdamsMeng LiBrian GreeleyMercé FalipBrian L. EdlowMeritxell Llorca-TorralbaBrian LoydMervyn J. EadieBrian R. ChristieMeytal WilfBrigitte KaufmannMiao LiBrijesh IyerMichael BackBrijesh SinghMichael BenderBrittany E. BlanchardMichael Christopher MelnychukBruna DenizMichael D. BerquistBruno AverbeckMichael EichingerBruno Del SetteMichael FalkensteinBruno Hebling VieiraMichael J. YoungBruno RossionMichael PalaciosBryanne BellovaryMichael ParsonsBulent AhishaliMichael PourfarByoung-Hee LeeMichael VitevitchByung-Hoon KimMichał BolaC. Geoffrey LauMichal KlichowskiÇağatay Murat YılmazMichal LavidorCailu LinMichal NovotnyCaitlin MitchellMichał StrzeleckiCălin I. ProdanMichal VostrýCalla M. GoekeMichela CandiniCalvin WuMichela FratiniCamilla BonaudoMichele AllegraCandace BurkeMichele CostanzoCándido InglésMichel-Edwar MickaelCankut ÇubukMichelle BoisvertCao DiMickael BonnanCara J. WestmarkMiguel A SimónCaren RandonMiguel LázaroCarine NguemeniMiguel LitaoCarla FarinhaMihai MusteataCarla MasalaMihai TomaCarla Strickland-HughesMikio HayashiCarlene MooreMikko Juhani HurmeCarlos Alfonso Tovilla-ZarateMilan TomaCarlos CepedaMilena ŚciskalskaCarlos Portugal-NunesMilena ŚwitońskaCarlos TrenadoMilena TomanicCarmelo CorsaroMilica BorovcaninCarmelo Luca SmeraldaMilica PešićCarmelo MillónMilorad DragićCarmen Daniela Quero CaleroMiloš VittoriCarmen Moret-TatayMin Chien TuCarmen Suárez-SerranoMingchung ChouCarol McNairMing-Feng WuCarole SousaMinghao DongCarolin KilianMinghao GongCaroline RanMing-Yuan MinCaroline SchnakersMin-Ho HanCaroline Walker-GleavesMinsuk KooCarsten SaftMinwoo LeeCaspar StephaniMiranda OcchioneroCassie S. MitchellMircea HuleaCatalina Márquez-VegaMireia FarrúsCatarina DurãoMiren AltunaCatarina GodinhoMiriam AkkermannCatarina RuaMiriam Annika VogtCaterina PalleriaMiriam Beatríz VirgoliniCathleen M. LutzMiriam BoppCatia NetoMiriam BragaCátia SeverinoMiriam Garrigós-PedrónCecile Bienboire FrosiniMirian Graciela Da Silva Stiebbe SalvadoriCecilia PerinMirko ManchiaCecilia PozziMiroslav VaverkaCecilia Zavala-TecuapetlaMirza PojskicCecilie RøeMisao NishikawaCelia B. HarrisMohamad EidCelia CruzMohamad El HajCeline SaulnierMohamed Abdelhafid KadriCesar CeballosMohamed AliCesare ZoiaMohamed Faisal ChevidikunnanChan Hee ParkMohammad Al-WardatChandra PrakashMohammad DaneshzandChang Eon ParkMohammad EtoomChang YaramothuMohammad Hassan A. NoureldineChang-Hoon ChoiMohammad Mehdi KafashanChanon NgamsombatMohammad ShahbakhtiChanpreet SinghMohammadreza ShalbafanChantal Delon-MartinMohammed DiykhChanung WangMohsen Mosayebi-SamaniCharisse B. PickronMohsen SoltanifarCharles F. GeierMojtaba VaismoradiCharles FinsterwaldMominur RahmanChatree Chai-AdisaksophaMonika HalickaChayoung KimMonika LeśkiewiczChen Hsiang-HanMonika MarcinkowskaCheng LyuMonika PudłoCheng Yin TanMonika RatajczakCheng-Chi LeeMonika SzeligaCheng-Fang YenMontserrat Corral VarelaChenghao ChenMonty SilverdaleCheng-Tsung HsiaoMoo-Ho WonCheng-Yang HuangMorena ShkodraCheng-Yu WeiMorgan GallazziniChenlu GaoMoria J. SmoskiCherie GreenMoritz HerzogCheryl GlazebrookMoses EkpenyongChian Ju JongMotoharu TakaoChiara PeciniMugtaba OsmanChiara SaraciniMuhammad NoumanChiara TobiaMuhammad Tariq SadiqChiara Valeria MarinelliMuhammad UmairChiara VillaMuhammad WaqasChien-Chin ChenMurali KolikondaChien-Huei HsuMurat GökdenChien-Hung LaiMyoung Won ChoChih Hung LoNachshon KoremChih-Cheng HuangNadine S. AguileraChih-Li LinNadinne RomanChih-Wei PaiNaeem HayatChih-Yang WangNagalingam RajakumarChintalapudi NaliniNagaraja S. BalakathiresanChiranji Lal ChowdharyNahian ChowdhuryChitra MandyamNaho KonoikeChiung-Mei ChenNaiara OzamizChong ChenNajeeb UllahChris CodeNaman VatsaChrista EinspielerNancy Monroy-JaramilloChristelle RobertNancy Pearl SolomonChristian ColletNaomi KakoschkeChristian Griñán-FerréNaosuke KameiChristian HydeNaoyuki TakeuchiChristian MoritzNapoleon Torres-MartinezChristian SchachtrupNarendra Nath JhaChristian TanislavNaresh Kumar HanchateChristiane S. HampeNastaran AhmadiChristiane SchwierenNatália Ferreira MendesChristie PanissiNatalia FilippovaChristina GillmannNatalia MadetkoChristina M. Vanden Bosch Der NederlandenNatalia MeirChristina ZhaoNatalia SuponevaChristine BrennanNatalia SzejkoChristine MohnNatalie N. NawarawongChristine SchiltzNatalya A. ShnayderChristine St LaurentNathalie Van Den BergeChristoph BledowskiNathan BeuclerChristophe DomingosNathan MuncyChristopher C. YoungNathan T. ZwagermanChristopher CorboNaveed Khan BalochChristopher David TurnbullNaveed Ullah KhanChristopher G. TarolliNavin Kumar OjhaChristopher R. CoxNawei SunChristos PapadelisNeamtu BogdanChristos SidiropoulosNeepa J. PatelChristos YapijakisNeeraj SoniChristy HomNegin MoradiChryssa BekiariNehal ElsherbinyChrystalina A. AntoniadesNeil Richard MillerChung-Min WuNela IlićChunki KimNell MaltmanChun-Po YenNelson CowanCinzia CecchettoNevin DarwishCinzia MarinaroNguyen Minh DucClair VandersteenNha NguyenClaire MitchellNicholas TrappClaire StockdaleNico AdelhoeferClaire-Marie RangonNicola ArmstrongClara Fernández-AlvarezNicola CavasinClara G. ZundelNicola MarottaClara SchollNicola MontanoClarissa Fantin CavarsanNicola MontemurroClaudia AltamuraNicola SalvadoriClaudia CantoniNicolas BurraClaudia Ferreira Da Rosa SobreiraNicolas LejeuneClaudia Iveth Astudillo-GarcíaNicolas StefaniakClaudia LermaNicole McLaughlinClaudia PisanuNienke H. Van DokkumClayton SwansonNihal ÇetinCollins Opoku-BaahNikolai FattakhovCompère LaurieNikolaj TravicaCong LiuNikolaos AntonakopoulosConor GillespieNikolaos DimitriadisConor J. WildNikolaos SmyrnisConrad PerryNikolaos ZygourisConstantin PotagasNikolas DovrolisConstantin TuleascaNina KaralijaConstantino Carlos Reyes-AldasoroNina WolskaCorentin J. GoslingNitika KumariCourtney GallenNitish V. ThakorCraig A. StockmeierNoomane BouazizCraig SurmanNoor SeijdelCristian DeanaNoriko SalamonCristian ScheauNorman M. SpivakCristiana Iulia DumitrescuNorman Scott LitofskyCristin WelleNounagnon AgbanglaCristina RosazzaNunzia PrencipeCrystell Guadalupe Guzmán PriegoNuria García-MarchenaCsaba HarasztosiNuria P Torres-AguilaCyrus MotamedNuttida RungratsameetaweemanaDagmar Schaffler-SchadenOksana ZinchenkoDalila BurinOlaia Lucas-JiménezDalin YangOlaia Martinez IglesiasDamien BénisOleg KovtunDamien GabrielOlga AndrianovaDan ChenOlga BoukrinaDan SongOlga IvanovaDana BadauOlga ShatokhinaDana CairnsOlga SysoevaDan-Alexandru SzaboOlga ValentimDandan ZhangOlive LennonDaniel BarbosaOliver BaumannDaniel BlackburnOlivier EspitiaDaniel C. CastroOlivier PierreficheDaniel HierOlivier Rémy-NérisDaniel J. ReidenbergOlli Hj GröhnDaniel SegenreichOlli TenovuoDaniel SeichepineOluwole O. AwosikaDániel VéghOmar CauliDaniel W. NixonOmar El HibaDaniela Carmen AbabeiOmar ParedesDaniela De BartoloOmer AydinDaniela LeccaOmer LinkovskiDaniela RiveraOmero Bendicto Poli-NetoDaniela TraficanteOmneya AttallahDaniele CorboOrlando G. P. BarsottiniDaniele PiscitelliOscar CrisafulliDaphna Mezad-KourshOscar WoolnoughDarin ZertiOshri AvrahamDario CalafioreOznur BilacDario SiniscalcoPablo Ezequiel Flores KanterDariusz KotlęgaPablo García-MirandaDariusz Wojciech MazurkiewiczPablo Usán SupervíaDarko ChudyPål RønningDarya Stanislavovna PereverzevaPallavi PatwariDave Saint-AmourPalma CiaramitaroDavid A. CunninghamPamela MayDavid A. PetersonPanagiotis KatrakazasDavid AmarantiniPanagiotis MallisDavid BakerPankaj AhluwaliaDavid BergeronPantelis LioumisDavid BrooksPaola AngelelliDavid Dodell-FederPaola FeracoDavid GrabliPaola GaldiDávid LíškaPaola PierobonDavid M. GreenberPaola RicciardelliDavid MandelbaumPaolo Antonino GrassoDavid Moreno MolinaPaolo BennaDavid ObertPaolo CampioniDavid PerpetuiniPaolo D'aquilaDavid R. M. A. HøjgaardPaolo FagoneDavid UttalPaolo ImmovilliDavid ZarkaPaolo MartellettiDavide CapponPaolo MazzoneDavide MaroccoPaolo MeneguzzoDawid GawlińskiPaolo SollaDawid StormanParisa GazeraniDaya Shankar GuptaPasqualina BuonoDayna Moya SepulvedaPatricia A. ShewokisDeanna GreenePatricia CaseyDebadatta DashPatricia DaviesDebo DongPatricia JusufDebolina BiswasPatricia Miranda AzpiazuDebora CiprianiPatrícia PiresDebora I. BurinPatricia Sánchez-Herrera BaezaDeborah Ann HallPatrick KwongDebra EhrlichPatrick LüningschrörDeepali S. JajuPatrick NeffDeepyaman DasPatrick SüβDeia GanayimmPatrizia FattoriDejan BudimirovicPatrizia TodiscoDelia CannizzaroPatrycja KleczkowskaDelia GagliardiPaul E. EngelhardtDemis BassoPaul E. ReedDenis Delisle RodriguezPaul LingorDenis PélissonPaul MearaDenise Nadine StephanPaul Michael RamirezDenise PergolizziPaul T. CirinoDespina MoraitouPaula Alexandra PostuDestiny E. O. AnyaiwePaula RegoDevesh PantPaulo Victor Sgobbi De SouzaDhanesh Sivadasan BinduPauravi J. GandhiDhanush HaspulaPavel KraikivskiDhrubajyoti ChowdhuryPaweł R. LataczDiana Angelika OlszewskaPaweł WańkowiczDiana KimonoPedro Gil-MadronaDiana R. PereiraPedro RoldánDidier DepireuxPeng FangDiego MinciacchiPeng-Jen ChenDieter ThomaPengmin QinDíez Revuelta ÁlvaroPengyu HongDimitrina MitevaPéter CsécseiDimitrios KasselimisPeter MachnikDimitrios PanagopoulosPeter RogeljDina ManettiPeter Van SchuerbeekDinesh BabuPeter ZhukovskyDinesh JillellaPetr MatoušekDinesh UpadhyaPetr VachataDing-Hau HuangPetronella KettunenDionysios TafiadisPeyman MirtaheriDipankar RoyPhanithi Prakash BabuDivya RadhakrishnanPhern-Chern TorDmitry A. SuplatovPhilipp KraussDmitry B. ZorovPhilippe GasqueDmitry LazurenkoPhilippe PeigneuxDmitry SergeevPhlip HaselDolors CañabatePier Nicola SergiDomenico De BerardisPierluigi ZoccolottiDominika GuzekPierpaolo AlongiDominika SkolmowskaPierre DecavelDominique DebannePierre DefresneDonald J. DeGraciaPierre GanderDonald PfaffPierre MouradDonato LiloiaPietro CacialliDong LiuPietro MaranoDong-Hyun KimPietro NazzaroDongrui MaPietro SpataroDonna M. PlattPing DongDora Luz OjedaPing SuDorota NieoczymPing-Tao TsengDorota Siwicka-GierobaPini LorenzoDouglas G. WalkerPinto-Almazán RodolfoDouglas MatthewsPiotr AlsterDouglas O'ShaughnessyPiotr ChmielarzDragana D. ProticPiotr PoznanskiDragica SelakovicPiotr PoznańskiDragos Catalin JianuPiotr WlaźDragoș Ioan TohăneanPlacido BruzzanitiDragos Paul MihaiPo-Cheng HsuDrew WeatherheadPo-Chun HsiehDrozdstoj St. StoyanovPommée TimothyDulce Maria MinayaPradoldej SompolDumitrescu CatalinPrajakt PandeDumitru CiolacPrakash KumarDursun KırbaşPranay SrivastavaDushad RamPrashanth Lingappa KukkleDusica PahorPrashanth PrabhuDuygu GözenPravesh Shankar GadjradjE. Begoña Garcia-NavarroPrit Benny MalgulwarEbrahim GhaderpourPriyankar PalEbrahim NorouziProsper N'GouemoEbru YıldırımPrzemysław DanekEce BayramPrzemysław DudaEckart ZimmermannPrzemysław KołodziejEddy J. DavelaarPushpa Raj JoshiEdna YamasakiQi CaiEdoardo Gioele SpinelliQi JinEdoardo MazzucchiQi LiuEdoardo MonfriniQiang LaiEduardo José Fernández RodríguezQiang ZhangEduardo LattariQianwei ChenEduardo ListikQifeng LiaoEdvard EhlerQingfang LiuEdward NęckaQinghui WangEdyta DziadkowiakQiuhai YueEfraín Santiago-RodríguezR. Weylin SternglanzEfrosini PapadakiRachel G. PizzieEfstratios-Stylianos PyrgelisRachid Ait AddiEiichi IshikawaRadosław ZajdelEiichi KumamotoRaeesa GupteEiichi NaitoRafael Torres-RosasEiki KimuraRafał CzajkowskiEirini OrovouRafał MorgaEitan GrossmanRafał StaszkiewiczEkaterina RudnitskayaRaffaela GiovagnoliEl Mostafa KalmounRaffaele NappoEleanor WilsonRaghavan GopalakrishnanEleftherios KavroulakisRahul K. KherElena Cecilia RoşcaRainer LeonhartElena ChikhirzhinaRainer MalikElena DruicaRajanikant PandaElena Escolano-PérezRajat Emanuel SinghElena Felipe-CastañoRajikha RajaElena FicoRajiv PathakElena KosenkoRajmohan DharmarajElena Pinero-PintoRajmohan RajmohanElena ZakharovaRaka CChem JainEleonora CatricalaRalf KetterEleonóra SpekkerRalph VollmannEleonora TopinoRaluca RăcăşanEleonora VirgilioRam Krishn MishraEleuterio SánchezRam SharmaElham FaghihzadehRamesh Kumar MuralimanoharElicia EstrellaRamesh Kumar PaidiEliete CaldeiraRamgopal KashyapElif Uygur-KucukseymenRami DiabElisa BaldinRamy AbdelnabyElisa BogossianRania ChristoforouElisa CairrãoRanjababu KulasegaramElisa CastaldiRaôul OudejansElisa MontanaroRaozhou LinElisa PaneroRaphael CiumanElisa VegezziRaphael FargierElisa VisaniRaquel FariaElisabet Llopart-saumellRaquel FaubelElisabet SerratRaquel Lozano BlascoElisabete P. SilvaRasa BarkauskienėElisabeth G. VichayaRatko LasicaÉlisabeth ThibaudeauRauf AhmadElisabetta BertelliniRaúl Quevedo-BlascoElise MarsanRavi Philip RajkumarElisete DiogoRavindra PeravaliElishai Ezra TsurRay KuEliza RussuRaymond A. ClarkeElizabeta B Mukaetova-LadinskaRaymond C. K. ChanElizabeth M. BrannonRaymond GoldsworthyElizaveta G. IazevaRaymond SalvadorElke EdelmannRazvan BoiangiuElliot MurphyRebecca BöhmeEllyn RileyRebecca GordonElmo P. PulliRebecca J. SharmanElvira MaranesiRebecca KerestesEman ToraihRedha AliEmanuele CassioliReena BapputtyEmile De KleineRegine BaderEmilia SitekRegis BobeEmilia Zabielska-MendykRégis BouquiéEmilie CardonReija AutioEmily C. MarcinowskiReinhard WerthEmin AltintasRemington L. NevinEmma ColamarinoRémy BationEmmanouil V. DermitzakisRenae StefanettiEnrico GiordanRenata ZajączkowskaEnrico MarcheseRenato GalzioEnrico RizzarelliRen-Fang ChaoEnrique García-MarcoReni KalfinErhan KavakbasiReza ZamaniEric C. FieldsRicardo Gómez-NietoEric J. WaldronRicardo KleinleinEric LarsonRicardo Salido RuizEric Van ExelRiccardo MancaErik BehringerRichard BaileyErin KamarunasRichard FunkErmanno QuadrelliRichard W. BohannonErnest TyburskiRichard WalshErwin Chiquete-AnayaRicky MuñozEsteban Obrero-GaitánRita CitraroEstefania NuñezRitesh RamdhaniEster NavarroRiwanti EstiasariEsther Martinez PastorRobert E. PykeEstrella RausellRobert Emmett KellyEthan KutluRobert HoerrEti Ben SimonRobert J. EnnisEttore SilvagniRobert SchnürchEugène A. RameckersRoberta BevilacquaEugenio Nelson CavallariRoberta CostanzoEunjin HwangRobertas DamaševičiusEunsu ParkRoberto ColluEustaquio AbayRoberto De La Torre-MartinezEva BagyinszkyRoberto Dell'AcquaEva MattRoberto Di MaioEva MöhlerRoberto GuidottiEva NiessenRoberto LicordariEvan AntzoulatosRoberto PalumbiEvan CampbellRoberto VagnettiEvangelia (Lila) DaskalakiRocío Montoya-PérezEvangelia AntoniouRocio Palomo-CarriónEvgenia GkintoniRodica BalasaEvgenia SitnikovaRodolfo SardoneEvgeny E. BezsonovRodrigo F. O. PenaEvgeny R. BojkoRodrigo RameleEwa Niechwiej-SzwedoRoger E. KelleyEwa Szczepanska-SadowskaRoger M. KrzyżewskiF. Scott HallRoksana MalakFabiana Maria Das Graças Corsi-ZuelliRoma SiugzdaiteFabien SauvetRomain SonnevilleFabio Cannas AgheduRomain TortuyauxFabio FerlazzoRomana VulturarFábio Filipe Fereira GarrudoRomeo Petru DobrinFabio SantacaterinaRon KedemFabio ScoppaRonald B. BrownFabrizia FestanteRonald BonnstetterFabrizio ParenteRonan TanguyFang WangRoni SettonFariborz GhaffarpasandRosa Grazia BellomoFarzad Vasheghani FarahaniRosa María Cabanas ValdésFatima YürekRosa María Giráldez-PérezFatma KolsiRosalba ParentiFausta LuiRosario J. MarreroFausto ViaderRossana RautiFaustyna ManikowskaRossella CaneseFauziah SulaimanRounak DeyFederica ManninoRoxana LucaciuFederico MorettiRoxanne BavarianFeiyan ChenRoy BeranFelipe Caamaño-NavarreteRoy G. BeranFelipe José AidarRubén Arroyo FernándezFelix Javier Jiménez-JiménezRuben Lopez-BuenoFelix Martin WernerRüdiger LandFelix NgRuhi TurkmenFengchao LangRuida HouFerath KherifRukmini Kandadai MridulaFernando Freitas GanançaRung-Tai LinFernando Muñoz-HernándezRuosen XieFernando Padovan-NetoRupert W. StraussFilippo BrighinaRupesh Kumar ChikaraFina Martínez-SolerRussell D'SouzaFiorella ColasuonnoRusty BarrettFiorenzo ContiRuth KrebsFiras KobeissyRuth McKenzieFlavia CardiniRuud L. Van Den BrinkFlavio Filippo AngileriRyan A. HenryFlavio LicciulliRyoki SasakiFletcher A. WhiteRyoma MorigakiFlorence ClavagueraRyouhei IshiiFlorence VorspanRyu WatanabeFlorent CarsuzaaS. M. Yasir ArafatFlorian ErgerS. RamkumarFlorian GesslerSabbatini MaurizioFlorian HeilmannSabina Barrios-FernándezFlorian KrauseSabina KrupaFlorian Ph.S FischmeisterSabine Jung-KlawitterFlorian SchmitzSabrina AristeiFlorian StielerSabrina GieseFloriana VolpicelliSabrina TurkerFotini BoufidouSachchida Nand RaiFotis TsetsosSadia NawazFrancesc GrausSadia ShakilFrancesc SolsonaSaeed SemnanianFrancesca Felicia OpertoSae-Yeon WonFrancesca Giulia MagnaniSahiti ChukkapalliFrancesca GrazianoSai-Fu FungFrancesca Luisa ConfortiSalar VaseghiFrancesca MontaroloSalvatore BonvegnaFrancesca PisanoSalvatore IaconoFrancesca TalpoSalwa El TawilFrancesca TrojsiSamad EsmaeilzadehFrancesca UbertiSamanta MazzettiFrancesco ComacchioSamantha BroadheadFrancesco CoreaSamantha C. PattonFrancesco Di GregorioSamir Al-AdawiFrancesco Di RussoSamir F. De A. CavalcanteFrancesco FischettiSamira ParhizkarFrancesco GaziaSampath VemulaFrancesco JanesSamson G. KhachatryanFrancesco OrziSamuel X. ShiFrancesco Paolo BusardòSanaullah SajibFrancesco Perono CacciafocoSandra AlhoyekFrancisco F. De-MiguelSandra BarbalhoFrancisco José Fernández-GómezSandra Denise PradoFrancisco Jose Garcia-MoroSandra McFaddenFrancisco MorenoSandra MooneyFrancisco Nieto EscámezSangmin LeeFrançois CossaisSani SaminuFrançois JamarSanjeet K. PandaFrançois TremblaySanjiban RoyFrank BoscoSantiago CepedaFrédéric GilbertSantiago J. BallazFredrik KarlssonSantosh GondiFriederike KlempinSara ColloroneFrigyes Samuel RaczSara CordesFukuda MitsuhiroSara GhiselliFulvio PlesciaSara InvittoGabriel G. De La TorreSara ParmigianiGabriela IoachimSara PerettiGabriela Oana BodeaSara PudasGabriela Rodriguez-ManzoSara SommarivaGabriela-Dumitrita StanciuSara SpadoneGabriele JanzenSara VarandaGabriele MelegariSarah Kristina BublitzGabriele NibbioSarah M. ClarkGabriele RussoSarah MasefieldGadi GoelmanSarah StevenageGagliardi Anna RobertaSaral DesaiGanesh M. BabulalSarath VijayakumarGarry KuanSarhang HamaGary MottolaŠarlota T. MesarošGautam PandeySarthak SinghalGengdi HuangSascha TopolinskiGeoffrey David ChampionSatja Mulej BratecGeoffrey LaforgeSatoshi HagihiraGeoffrey SchwartzSatoshi MatsuoGeorge P. KostyukSatoshi TakahashiGeorge PavlidisSatoshi YokoyamaGeorge S. KorresSatwant KumarGeorge SpanoudisSatya Murthy TadinadaGeorge UmemotoSaurabh JhaGeorgia AngelopoulouSaurabh PandeyGeorgina LynchSawsan A. ZaitoneGeorgios AlexandrakisSayyed Ali SamadiGeorgios GeorgiouScott CoussensGeorgios NarosScott SinnettGeorgios SioutasScott SpreatGerd Fabian VolkSe Jin ParkGerd WagnerSean MeehanGergely FeherSebastian BeckerGergely ZachárSebastian CozmaGermano GallicchioSebastian GłowińskiGesa SchaadtSebastiano Alfio TorrisiGessynger Morais-SilvaSebastien RimbertGhorban TaghizadehSebastien RoyerGhulam GilanieSe-Chan KimGhulam Md AshrafSeckin AkgulGiacinto BarresiSedighe KarimzadehGiacomo NovembreSeema NagpalGiada LettieriSeema PrasadGian Marco DumaSeifollah GholampourGianluca CampanaSelçuk GüvenGianluca EspositoSeok Joon WonGianluca LavancoSeolwoo ParkGianluca MalatestaSeong Soo A. AnGianluca SusiSeong-Ho KohGiia-Sheun PengSerena DattolaGil SuzinSerge BrandGilberto GalindoSergei G. GaidinGilberto Pérez-SánchezSergei TuginGillian RiordanSergey MalkinGioacchino TedeschiSergey MosolovGioele GavazziSergey V. RoussakowGiordano D'ursoSergio CorvinoGiorgi KuchukhidzeSergio FrumentoGiorgia CommitteriSerkan YílmazGiorgia VaralloSeung-Jong KimGiorgio PalandriSeyed Mostafa KiaGiorgio VallortigaraSeyed Reza ShahamiriGiovanna FurneriShahin NasrGiovanni AnobileShalini DograGiovanni AssenzaShalisah SharipGiovanni FanniShamik ShahGiovanni FedericoShancheng BaoGiovanni FerraraShangru LyuGiovanni Freitas GomesShanlin KeGiovanni GaleotoShaoxun WangGiovanni MirabellaSharon AshGiovanni MuscasSharzad MohseniGiovanni RuoppoloSheikh K. Tahajjul TaufiqueGisela StoltenburgSheikha MajidGiulia AbateSheila BlackGiulia BallardiniSheila MansouriGiulia BerzeroShelli KeslerGiulia CartocciSheng-Nan WuGiulia MenculiniSherief GhozyGiulia TorrominoSherryse CorrowGiulia VarottoShiek S. S. J. AhmedGiulio BertaminiShikha Talwar BassiGiulio BiondiShing-Hwa LiuGiulio ContemoriShintaro SukegawaGiulio GabrieliShirley WyverGiulio SenesShivani D. RangwalaGiuseppe BarisanoShivani SoniGiuseppe BiaginiShoko HaraGiuseppe IatíShokoufeh Monjezi KouchakGiuseppe La RoccaShovan NaskarGiuseppe LanzaShuhsien ChuGiuseppe LiscoShun-Fa YangGiuseppe SciamannaShunta MaedaGiuseppe TalaniShuo ZhaoGiuseppe VaroneSicong LiuGiuseppina CatanzaroSiddhita D. MhatreGleb V. TcheslavskiSidney P. KuoGlenn YamakawaSigbjørn LitleskareGniewko WięckiewiczSigrid TheunissenGolbarg MehraeiSilvana GalderisiGonca Gokce Menekse DalverenSilvia A. OddoGonçalo DiasSilvia PrimativoGonzalo Emiliano Aranda-AbreuSilvia SeghezziGonzalo Varas-DíazSilvia ToricesGordon SloanSilvio IontaGraciela Catalina Alatorre-CruzSilviu MatuGraciela Padilla-CastilloSimon Morand-BeaulieuGraham M. WinstonSimona LattanziGrant D. SearchfieldSimona MitevaGrazia GalleriSimona PichiniGraziella MangoneSimona SacchiniGregor BroessnerSimone BattagliaGregory FonzoSimone Di PlinioGregory KróliczakSimone Dos Santos BarretoGreta MainieriSimone RossiGrzegorz GrześkSimone SilvaGrzegorz WysiadeckiSina ZoghiGueesang LeeSindhuja Tirumalai GovindarajanGuido Marco CicchiniSireesha ManneGuillaume ThibaultSiti Rafidah YusofGuillermo PlazaSiva DurairajanGulmira BekmanovaSivaraj Mohana SundaramGunter KreutzSivaraman NatarajanGuobin XiaSiwan SeamanGuofang ShenSiyi ChenGuoli ZhengSiyuan HuangGustavo Fernández-PajarínSjoerd J. H. EbischGustavo GasaneoSławomir MańdziukGuy HansSmadar Cohen-ChenGvozden RosicSneha VivekanandhanGyorgy LurSofia StraudiGytė DamulevičienėSofya MorozovaHaewon ByeonSofya P. KulikovaHaihong ZhangSohail MumtazHaipeng LiuSoheil KeshmiriHaiqiu HuangSoichiro TakamiyaHaitao LiuSokol TrunguHamid MukhtarSolveig Lægreid HaugerHamid Reza MaratebSomayeh AghanavesiHamoon ZohdiSonam MittalHan ZhangSondra T. BlandHana NůskováSonia MeleHanani Abdul MananSonu SinghHang ZengSoo-Eun ChangHannah PazderkaSophia CharitouHany Hamdy ArabSophie DuportHarikesh DubeySophie RijnenHariklia ProiosSotirios ApostolakisHarikumar RajaguruSpyridon ChavlisHarsh DeoraSpyridon DoukakisHarshani WijerathneSpyridon TsiourisHarshit S. ParmarSricharan S. VeeturiHartmut VatterStanislas LagardeHarun NoristaniStefan A. FrischHarvey R. FernandezStefan DuschekHarvey SarnatStefan MihaicutaHasan Al-NashashStefan SchmidtHatice TankisiStefan SchobHaydèe Fiszbein WertznerStefan SchoisswohlHéctor García- LópezStefan SchweinbergerHedde ZeijlstraStefania ScaliseHeechul YoonStefano CasalottiHelen McaneneyStefano Maria PriolaHelena Maria Tannhauser BarrosStefano MusardoHelmut Schrom-FeiertagStefano RamatHemant PrajapatiSteffi M. UrbschatHendrik BerthStella Felix LourencoHenggui ZhangStella María Fuensalida NovoHenk Maria KoningStephan LauxmannHenning BoeckerStéphane PerreyHerlinda Bonilla JaimeStephanie DeLucaHero P. WitStephanie DuguezHideaki KawabataStéphanie LefebvreHideaki ShigaStephanie MaloneHideki NakanoStephanie WongHidenori SuganoStephen Andrew VernonHidetoshi KomatsuStephen LippiHideyuki J. MajimaStephen SkalickyHiide YoshinoSteven G. KovenHilary MossSteven PanHipolito NzwaloStoyan PopkirovHiran ThabrewSu YangHirendra Nath BanerjeeSuan LeeHiroaki NekiSudhakar TummalaHiroaki TaniguchiSudhirkumar YanpallewarHiroshi IrisawaSudip SenHiroshi NoguchiSue BuckleyHirotaka GimaSufang LiuHisham BahmadSukeun JeongHiu Chuen LokŞükrü ÇağlarHolger A. RamboldSuleman LazarusHolger HillSuleyman KaplanHolman TseSuman ChowdhuryHomira BehbahaniSumitha Rajendra RaoHonghe LiuSunan LiHong-Hsiang LiuSunny Malhotra SareenHongsun GuoSuparerk JanjarasjittHope Sparks LancasterSuravajhala PrashanthHossein Tabatabaei-JafariSurendra RajpurohitHo-Won LeeSurjeet SinghHsien-Tsai WuSurujpal TeelucksinghHsin-An ChangSusana Muñoz ManiegaHsiuying WangSusana SilvaHu NgSuzanne HanserHuaishuang ShenSven MånssonHuali WangSvetlana AidagulovaHubertus AxerSwagata GhatakHubertus HimmerichSylvère StörmannHugo CordeiroSzidónia LefkovitsHuiming LuTakahiko KoikeHung-Chuan PanTakahiro YamauchiHyungon LeeTakanori OhnishiHyunji KimTakayoshi UbukaHyunjoong KimTakehiro UdaHyunWook ParkTakemichi FukasawaI-Chien WuTakeo MukaiIgnacio LijarcioTakeshi HiuIgnasi Navarro SoriaTakeshi UnokiIgnazio Gaspare VetranoTakuma InagawaIgor AleksanderTali WeissIgor GruićTamal SadhukhanIke De La PeñaTamar BasishviliIlaria BufalariTamara GaloyanÍlknur ÖzenTamás Ollmannİlknur Sur ErdemTang SanIlona Rubi-FessenTanja Grubić KezeleIman AganjTanja Schmitz-HübschIman BeheshtiTanu WadheraIman TavassolyTanya J. LehkyIn Seok MoonTanya SaraiyaIna Reić-ErcegovacTanzil Mahmud ArefinIndika Neluwa-LiyanageTao LiuIndrani PoddarTao XieInes WolzTatiana AntipovaInken HöllerTatsat PatelInmaculada LeónTatsuhito HimenoIoannis LiampasTatsuo SawadaIon BeratisTatsuya YamamotoIona NovakTayebeh KazemiIqram HussainTejeshwar RaoIrena Baranowska-BosiackaTelma AlmeidaIrena Brčić KaračonjiTelmo Raul Aveiro-RóbaloIrena NalepaTeneille Emma GoftonIrene Cortés-PérezTeng J. PengIrene ValoriTerence MoriartyIrina Iuliana CostacheTeresa FemeníaIrina MäurerTerry LichtorIrina MocanuTeru KamogashiraIrina NovikovaTestusko KasaiIrna Elina RidzwanTeyu LinIsa ZappulloThalía FernándezIsabel Corrales-GutiérrezThanuja DharmadasaIsabel Fernández-PérezTheng Choon OoiIsabelle M. RossoTheodora PapamitsouIva LukicThiago P FernandesIván Herrera-PecoThijs Van Der ZijdenIván Padrón GonzálezThomas A. BuseyIvana KraljevicThomas C. ThannickalIvana NovakovicThomas HollandIvarne L. S. TersariolThomas J. RossIzabela ZakrockaThomas KleinsorgeJ. Andrew OnesimuThomas LachmannJ. Francis BorgioThomas LindnerJ. Matt McCraryThomas NyffelerJ. Simon WiegertThomas PerreaultJacek BucznyThomas SchmidtJacek FurtakThomas TannouJacek WilczyńskiThomas WeltonJack L. FollisThomas WolfJack ParkerTibor GuzsvineczJacky GangulyTijana BojićJaclyn Hennessey FordTilmann H. SanderJaclyn StephensTim HodgsonJacob PaulTimothy BoergerJacopo FalcoTina Dam KristensenJacopo HarzoniTingxin ZhangJacopo Junio Valerio BrancaTizian RosenstockJacopo LanzoneTiziana CiarambinoJacques PyTiziana CollettiJae Meen LeeTiziana QuartoJagdeep KaurTjeerd V. Olde ScheperJaisan IslamTobias WeissJakob PietschnigTolga SoykanJakub NalepaTolga ZamanJames D KeanTomáš KrejčíJames HentigTomaso VecchiJames O'CallaghanTomasz FrączykJames P. BennettTomasz KuligowskiJames PowersTomaž SnojJames W. SonneTommaso BocciJamie WardTommaso CalloviniJamir Pitton RissardoTommaso CostaJan BakosTommaso ErcoliJan BroggerTommaso Francesco GalbiatiJan CendelinTommaso VerdolottiJan ChrastinaTomoki NakamuraJan EggerTomoko LeeJan FousekTomoyuki MaruoJan GramsbergenTonhajzerová IngridJan J. ZebrowskiTor EndestadJan MehnertTorbjörn LedinJan StoopToru TaguchiJana TegelbeckersToshihiko TashimaJanani ParameswaranToshio HigashiJanet DubinskyToyoaki OhbuchiJanez RavnikTracy M. CentanniJannik PrasuhnTrang NguyenJared B. SmithTrevor ArcherJaromír MyslivečekTrevor CrawfordJaroslav KacetlTrevor GersonJaroslava Paulasova SchwabovaTri Niswati UtamiJarosław CholewaTriantafyllos DidangelosJasmina IsakovićTrieu NguyenJasneek K. ChawlaTushar IssarJason John LabuschagneTzu-Yu HsuJason S. RadowskyUdaiyappan JanakiramanJaume VivesUdit SaxenaJavier García-OrzaUehara TomokoJavier MartínUlf C. SchneiderJavier SantabarbaraUlf Jensen-KonderingJawza AlsabhanUllrich WüllnerJay L. AlbertsUlrich PomperJean A. RondalUlrike KumpfJean Marie BillardUma ChandrachudJean-Luc GaiarsaUnsong OhJean-Marc BarretUrszula Sajewicz-RadtkeJean-Marie AnnoniUsman GhafoorJeanne GalléeUte GschwandtnerJeannette Hübener-SchmidUte LindauerJean-Paul RichaletUwe IlgJean-Yves ChattonVáclav VencovskýJed MeltzerVaclava PioreckaJeffrey BurgdorfVadim GrubovJeffrey Hugh GambleVahab Youssof ZadehJeffrey R. GagneVaidotas StankevičiusJehad AliVaishali AggarwalJehuda SolemanValentina CerratoJelena KosticValentina LatinaJelena MartinovicValentina MoroJennifer H. SuorValentina MosienkoJennifer LehmannValentina PallottiniJennifer M. McDermottValentina Rosa LaganàJennifer VermilionValeria CostanzoJeremy T. NelsonValeria LucchinoJeroen A. Van WaardeValeriia DemarevaJerónimo González-BernalValério Antonio Pamplona SalomonJesper Just FabriciusVan Eimeren ThiloJesse GaleVandana BhattacharjeeJessica LowethVandana VarmaJessica Magid-BernsteinVanessa Arán-FilippettiJessica MeersVaradaraya Satyanarayan ShenoyJesús María López-ArrietaVasileios KokkinosJesus PastorVasilije StambolijaJewoong MoonVassiliy TsytsarevJiahe LiVeena DesaiJiamei HouVelia D'AgataJianguo ZhangVenkata Chaitanya ChirumamillaJiaqi ZhangVenkatakrishnan RengarajanJijia WangVera GuttenthalerJimmy LiVerner KnottJimmy StehbergVeronica Alexandra AntipovaJing PangVeronica L. TimbersJing ZhaoVerónica Mäki-MarttunenJin-Huyk ParkVeronica MuffatoJinming HanVéronique DelvenneJinn-Rung KuoVeronique Deschodt-ArsacJinyu LiVéronique Marie AndréJiří KantorVessela KrastevaJishnu KrishnanVictor KallenJitka BuskovaVíctor M. RiveraJitka MarenčákováVictor RiveraJitka VeldemaVictoria C. P. KnowlandJitse P. Van DijkVictoria Gómez-MurciaJoana AchaVigneswaran VeeramuthuJoana Barroso MonteneiroVijai KrishnanJoana CabralVijay KumarJoana DamásioViktoria SarafianJoana DuarteVille LeinonenJoanna GruszczyńskaVinayKumar IdikudaJoanna KostkaVincent Breton-PrivencherJoanna SikoraVincent PonsJoão Gama MarquesVincente Javier Clemente-SuarezJoão MartinsVincenzo Di StefanoJodie Davies ThompsonVincenzo NasilloJoelle ChabryVinita BharatJoeri K. TijdinkVirginia FancelloJohan KlebergVirginie MoulierJohanna SeifertVirginie NeirinckxJohannes VorwerkVishwanath SankarasubramanianJohn A WoodsVitalie LisnicJohn Amson CapitmanVitor ParolaJohn D. PapatriantafyllouVivian CiaramitaroJohn Dirk Vestergaard NielandViviana MucciJohn F. ConnollyVladimir BaščarevićJohn GreenVladimir V. BezuglovJohn K. YueVladislav ToronovJohn KomarVolker A. CoenenJohn Shelley-TremblayWalter BusuttilJohn SteinWalter MaetzlerJohn ThomasWalther BildJohnny PaduloWanchun SuJo-Hsuan WuWanich SuksatanJolanta DorszewskaWantana SiritaratiwatJolanta MasiakWarawoot ChuangchaiJolanta StrzeleckaWei ShaoJonas VibellWei WangJonatan LefflerWei ZhangJonathan GraingerWei-Chieh LeeJoon W. ShimWeiguo LiJoost Van Der VorstWeihua WangJordan FollettWei-Ju ChangJordan PierceWeiyan YinJordi Maitas-GuiuWeiying DaiJordy ThielenWen Han TongJorge Diogo Da SilvaWenke MöhringJorge M. LaínsWenqiang FanJørgensen Louise M.Wenzhen CaoJori AkimotoWhitney E. EnglandJose A. PerianezWiktor JedrzejczakJosé A. SáezWiktoria WojniczJosé Ángel ArmengolWilbert J. HeeringaJose Antonio Lopez-EscamezWilliam BergJose C. AdsuarWilliam CaudleJosé Carmelo AdsuarWilliam R. ReedJosé Enrique Moral-GarcíaWilliam XuJosé Hernández OrtegaWinko W. AnJose Jonatan Cortés-MartínWioletta PawlukowskaJose M. Rodriguez FerrerWladimir Rafael BeckJosé Manuel CimadevillaWładysław LasońJose Paulo Marques Dos SantosWłodzimierz ŁuczyńskiJosefa Gonzalez-SantosWolfgang GrisoldJosep PetchaméWolfgang SchadenJoseph K. CarpenterWon Hee LeeJoseph R. DuffyWoon-Man KungJosephine MollonWoo-Ri ChaeJoshua LittleXavi Suárez-CalvetJovana BjekićXenophon ZabulisJoy MitraXiangyu RenJoyce SietteXianqi LiJuan Antonio Valera-CaleroXianwei CheJuan Carlos Martínez-GutiérrezXiaodong MaJuan Castillo-MartinezXiaokuang MaJuan Crespo MariñoXiaoli GuoJuan Evaristo Callejas-JerónimoXiaoming DuJuan Francisco Vázquez CostaXiaoming JiangJuan Luis Sánchez GonzálezXiaoqian YuJuan M DominguezXiaosu HuJuan Manuel DoderoXiaoyu SongJuan P. RomeroXin ZhangJuan Pedro Martínez RamónXindong MaJubayer A. HossainXuan HaJudit OláhXuan MaJudit ZsugaXuesong (Andy) GaoJue ZhouXueying WangJulia E. Marquez-ArricoYaara Endevelt-ShapiraJulia Jones HuyckYael Doreen LewisJulia LischkaYang HeJulián Albarrán JuárezYang YuJulian CheronYang ZhangJulian ConradYanhui LiaoJulian Paul KeenanYannis KaramanosJulian ReiserYarden GliksmanJulian S. RechbergerYarema B. BezchlibnykJuliana Marisol Godinez-RubiYasuharu KoikeJuliana MoreiraYasuhiko HayashiJuliana Novo GomesYasuhiro KuroiJulie LissYasuko TanakaJulie ScorahYasunari MatsuzakaJulien Saint-PolYasuro AtojiJuliet Haarbauer-KrupaYaswanth KuthatiJulieta Domínguez-SoberanesYeon-Mo YangJulio Plaza DíazYeou-Jiunn ChenJulio Vicente Figueroa-MillánYeşim Yaman AktaşJulio-César De La Torre-MonteroYi ChenJulius HöhneYi LuanJumana SalehYicheng LongJun Hui JeongYi‐Chia WeiJun Jie Benjamin SengYifei HeJun Neng RoanYiheng TuJun SoneYijing ZhouJun XieYijun PanJunaid AnsariYingying WangJunchao TongYinliang LinJunhyung KimYixiang DengJunyeon WonYogananda S. MarkandeyaJustine BailleulYogi Tri PrasetyoJustyna GerłowskaYoko SuzukiJustyna Sarzyńska-WawerYongcong ShaoJustyna ŚwidrakYoram ShirJuvenal Rodríguez-ReséndizYorito HattoriJyoti MishraYoshifumi MizunoJyoti SihagYoshihiro NodaKabirullah LutfyYoshiro ItoKah Hui WongYoshitaka OkuKaibao NieYosuke MotekiKaila L. StipancicYoucef BouchekiouaKalpna GuleriaYoung Beom SeoKalyan Brata BhattacharyyaYoung Ho KohKalyani ChaubeyYoungeun KimKamalesh ChakravartyYoung-Zoon KimKamila RachubińskaYouye XieKamila RasovaYu ChenKamila Zglejc-WaszakYu ZhangKamilla Rún JóhannsdóttirYuanhao YangKamrad Khoshhal RoudposhtiYuankai QiKamyar MoradiYuanmay ChangKang Min ParkYuchen XuKang-Ming ChangYuchuan DingKanjana UnnwongseYudai YamazakiKara JohnsonYu-Fang WangKarim ReFaeyYuhei NishimuraKarin HeidlmayrYu-Hsiang KuanKarine MichaudYuichiro ShirotaKarl-Michael SchebeschYulia K. KomlevaKarsten WittYun-Hee KimKartik K. IyerYunjung KimKatarína BabinskaYushin TakemotoKatarina KouterYu-Shu HuangKatarina RukavinaYuuichi HoriKatarina VukojevicYuyang LuoKatarzyna BobrowiczYu-Ying ChuangKatarzyna GóralskaZachary IrwinKatarzyna HarezlakZahra AshkavandKatarzyna JankowiakZahra HooshyariKatarzyna MarkiewiczZargari Marandi RamtinKatarzyna NowomiejskaZarin ZainulKatarzyna WyskidaZbigniew AdamiakKate M. ChittyZbigniew IzdebskiKatharina BeyerZdzisław SroczyńskiKatharina LutzZekiye AltunKatharina SchimmelZenon MariakKatherine W. TurkZetian YangKathleen MonahanZhangyu ZouKathrin KollndorferZhaoqian TengKathrine Jáuregui-RenaudZhemeng WuKathryn ConnaghanZhenglin GuKathryn E. GustafsonZhijun PuKathryn LenzZhongfei BaiKatlyn BrownŽiga KozincKatrin RauenZirui HuangKatrina McFerranZohar EviatarKatsunori FuruyaZohreh MousaviKatsuya SakaiZsuzsanna MihályKaustuv GanguliZsuzsanna RecsanKavita SinghZubeda SheikhKazu AmimotoZuzana RošťákováKazuhide ShimizuZuzana ŠkodováKeigi Fujiwara



